# Schistosomiasis in non-endemic areas: Italian consensus recommendations for screening, diagnosis and management by the Italian Society of Tropical Medicine and Global Health (SIMET), endorsed by the Committee for the Study of Parasitology of the Italian Association of Clinical Microbiologists (CoSP-AMCLI), the Italian Society of Parasitology (SoIPa), the Italian Society of Gastroenterology and Digestive Endoscopy (SIGE), the Italian Society of Gynaecology and Obstetrics (SIGO), the Italian Society of Colposcopy and Cervico-Vaginal Pathology (SICPCV), the Italian Society of General Medicine and Primary Care (SIMG), the Italian Society of Infectious and Tropical Diseases (SIMIT), the Italian Society of Pediatrics (SIP), the Italian Society of Paediatric Infectious Diseases (SITIP), the Italian Society of Urology (SIU)

**DOI:** 10.1007/s15010-023-02050-7

**Published:** 2023-07-07

**Authors:** Agnese Comelli, Camilla Genovese, Federico Gobbi, Gaetano Brindicci, Susanna Capone, Angela Corpolongo, Verena Crosato, Valentina Dianora Mangano, Rosalia Marrone, Maria Merelli, Marco Prato, Carmen Rita Santoro, Salvatore Scarso, Elisa Vanino, Valentina Marchese, Spinello Antinori, Claudio Mastroianni, Annibale Raglio, Fabrizio Bruschi, Andrea Minervini, Daniele Donà, Silvia Garazzino, Luisa Galli, Andrea Lo Vecchio, Andrea Galli, Gabriele Dragoni, Claudio Cricelli, Nicola Colacurci, Enrico Ferrazzi, Annalisa Pieralli, Antonio Montresor, Joachim Richter, Guido Calleri, Alessandro Bartoloni, Lorenzo Zammarchi

**Affiliations:** 1 Infectious Diseases Unit, Foundation IRCCS Ca’ Granda Ospedale Maggiore Policlinico, Milan, Italy; 2 Department of Biomedical and Clinical Sciences, University of Milan, Milan, Italy; 3 II Division of Infectious Diseases, ASST Fatebenefratelli Sacco, Luigi Sacco Hospital, Milan, Italy; 4 Department of Infectious-Tropical Diseases and Microbiology, IRCCS Sacro Cuore Don Calabria Hospital, Negrar, Italy; 5 University of Brescia, Brescia, Italy; 6 AOU Consorziale Policlinico di Bari, Infectious Diseases Unit, Bari, Italy; 7 Department of Infectious and Tropical Diseases, University of Brescia and ASST Spedali Civili of Brescia, Brescia, Italy; 8 National Institute for Infectious Diseases ‘Lazzaro Spallanzani’ (IRCCS), Rome, Italy; 9 Department of Translational Research, N.T.M.S, Università di Pisa, Pisa, Italy; 10 Programma Di Monitoraggio Delle Parassitosi e f.a.d, AOU Pisana, Pisa, Italy; 11 National Institute for Health, Migration and Poverty, Rome, Italy; 12 Azienda Sanitaria Universitaria del Friuli Centrale, Udine, Italy; 13 National Center for Global Health, Istituto Superiore di Sanità, Rome, Italy; 14 Unit of Infectious Diseases, Ospedale “Santa Maria delle Croci”, AUSL Romagna, Ravenna, Italy; 15 Department of Infectious Diseases Epidemiology, Bernhard Nocht Institute for Tropical Medicine (BNITM), Hamburg, Germany; 16 III Division of Infectious Diseases, ASST Fatebenefratelli Sacco, Luigi Sacco Hospital, Milan, Italy; 17 Department of Public Health and Infectious Diseases, Sapienza University of Rome, Rome, Italy; 18 Committee for the Study of Parasitology of the Italian Association of Clinical Microbiologists (CoSP-AMCLI), Milan, Italy; 19 Department of Experimental and Clinical Medicine, University of Florence, Florence, Italy; 20 Department of Urology, University of Florence, Florence, Italy; 21 Division of Paediatric Infectious Diseases, Department for Women’s and Children’s Health, University of Padua, Padua, Italy; 22 Paediatric Infectious Disease Unit, Regina Margherita Children’s Hospital, A.O.U. Città della Salute e della Scienza di Torino, Turin, Italy; 23 Infectious Diseases Unit, Meyer Children’s Hospital, IRCCS, Florence, Italy; 24 Department of Health Sciences, University of Florence, Florence, Italy; 25 Department of Translational Medical Sciences, Paediatric Infectious Disease Unit, University of Naples Federico II, Naples, Italy; 26 Gastroenterology Research Unit, Department of Experimental and Clinical Biomedical Sciences “Mario Serio”, University of Florence, Florence, Italy; 27 Health Search–Istituto di Ricerca della SIMG (Italian Society of General Medicine and Primary Care), Florence, Italy; 28 Department of Woman, Child and General and Specialized Surgery, University of Campania “Luigi Vanvitelli”, Naples, Italy; 29 Department of Woman, New-Born and Child, Fondazione Istituto di Ricovero e Cura a Carattere Scientifico Ca’ Granda Ospedale Maggiore Policlinico, Milan, Italy; 30 Ginecologia Chirurgica Oncologica, Careggi University and Hospital, Florence, Italy; 31 Department of Control of Neglected Tropical Diseases, World Health Organization, Geneva, Switzerland; 32 Institute of International Health, Charité Universitätsmedizin, Corporate Member of Freie und Humboldt Universität Berlin and Berlin Institute of Health, Berlin, Germany; 33 Amedeo Di Savoia Hospital, ASL Città di Torino, Turin, Italy; 34 Infectious and Tropical Diseases Unit, Careggi University Hospital, Florence, Italy

**Keywords:** Schistosomiasis, Katayama syndrome, Migrant health, Travel health

## Introduction

Human schistosomiasis is a chronic parasitic disease caused by trematode worms of the genus *Schistosoma*. It affects people living in tropical and subtropical regions where approximately 780 million people are at risk[1].

Sub-Saharan Africa (SSA) is the main hotspot for schistosomiasis, with roughly 90% of the global cases and ∼ 300 000 annual related deaths [2].

Europe receives a constant flow of migrants, among them, a significant proportion comes from schistosomiasis-endemic countries. Over the past 6 years, Italy has received an unprecedented number of migrants of almost 60,000 people per year, a high number even considering the lower migration flow during the COVID-19 pandemic[3].

As a result, the number of people in Europe at risk of having chronic schistosomiasis is potentially high considering migrants from schistosomiasis-endemic countries and people traveling for business or leisure from non-endemic to endemic countries.

The most recent literature estimates that nearly 24% of SSA migrants test positive for anti-Schistosoma spp. Seroassays [4] and diagnosis of schistosomiasis is not unusual among travellers[5, 6]. Non-endemic countries need to implement simple and practical guidelines to quickly identify acute and chronic disease, correctly staging its severity, efficiently treat and follow up the infected individuals.

Unfortunately, rigorous evidences on schistosomiasis screening, diagnosis and management are often scarce or insufficient.

Considering those limits, this document is based on a broad review of the available literature performed by members of a multidisciplinary panel belonging to the Italian Society of Tropical Medicine and Global Health (SIMET). The study group took advantage of the scientific support of national and international experts and other Italian scientific societies which are involved in schistosomiasis management.

## Methods

The objective of this document has been to develop handy and clinically relevant recommendations on schistosomiasis screening, diagnosis and treatment useful for a broad range of health care providers such as general practitioners and paediatricians, infectious disease and travel health specialists and other specialized physicians potentially involved in the therapeutic or diagnostic phase (e.g., general practitioners, hepatologists, urologists, gastroenterologists, gynaecologists).

Major recommendations have been validated through a Delphi consensus-seeking procedure involving fourteen internal experts, that participated in Delphi procedures and four external experts who collaborated in developing statements and reviewed evidence summary [7].

External advisors have been selected among the international scenario of schistosomiasis experts to cover different areas: policy, clinical management, radiologic expertise and parasitology.

The Delphi consensus process uses three rounds to assess the level of agreement on recommendations and facilitate their refinements.

The experts were asked to assign a score on a 9-point Likert scale to each proposed statement of the recommendations, where 1 represented the lowest level of agreement and 9 the highest. If the panellist assigned a score < 9, he/she was asked to explain the reason of his/her mark and to suggest a correction while providing one or more bibliography references to support it.

Each round was conducted via an online survey and consensus was reached when at least 70% of the scores were in the range 7–9 on the Likert scale.

Two rounds were needed to reach consensus for all the statements proposed. The detail of the Delphi process and scores are presented in Supplementary table 1.

These consensus recommendations are not intended to cover all the clinical presentations of *Schistosoma* spp. (e.g., cercarial dermatitis, neuroschistosomiasis, ectopic infection in lungs, spleen etc.) mainly because of the rarity of such observation and, consequently, the challenge of drawing recommendations in settings where a consistent body of literature is lacking.

BOX 1 human schistosomes, exposure and geographical distribution WHO estimates that approximately 230 million people have schistosomiasis worldwide and 90% of them live in Africa. *Schistosoma* spp. Is also endemic in parts of Latin America and Asia [8], and autochthonous cases were reported in Corsica, France and Almeria, South-East Spain [9, 10].

There are five main pathogenic species that affect humans: *S. mansoni, S. japonicum, S. intercalatum, S. guineensis* and *S. mekongi* which cause hepato-intestinal schistosomiasis and *S. haematobium* which affect the uro-genital tract [11].

Moreover, hybrids between different species that are closely phylogenetically related and share the same intermediate host may result from co-infection in the same definitive host (e.g., *S. haematobium* and animal) [12].

Infection occurs when individuals contact freshwater bodies infested with cercariae released by specific intermediate host snails (genus *Bulinus* spp, *Biomphalaria* spp, *Oncomelania* spp, *Neotricula* spp) previously infected through contamination of water by stool or urine of individuals with schistosomiasis releasing parasite eggs with their excreta.

Transmission occurs through the penetration of intact skin by *Schistosoma* spp. cercariae. Acute symptoms of cercarial dermatitis may be associated with the infection event, but this is unspecific since it may also derive from contact with animal Schistosoma species which do not result in infection.

Later on, cercariae mature into adult worms that migrate to the mesenteric venous system or the venous plexus of the bladder where the females lay their eggs. Rarely, some adult worms may end up before the target organ. Some of the eggs are eliminated via stool or urine, others are trapped in body tissues causing chronic inflammation and fibrosis.

BOX 2 definition of Katayama syndrome and chronic schistosomiasis **Katayama syndrome**

POSSIBLE

Presence of one or more of the following signs or symptoms: fever, cough, hepato/splenomegaly and rash (generally urticarial) in individuals who have lived in or travelled to endemic countries during the previous 3 months; particularly if they report contact with freshwater.

PROBABLE

Possible Katayama syndrome.

AND

Positive *Schistosoma* serology and/or eosinophilia and/or evidence of nodules and ground-glass areas on X-ray or CT scan.

PROVEN

Probable Katayama syndrome.

ANDat least one among the following:

- Eggs in stool, urine or tissue biopsy;
- Detection of *Schistosoma* DNA by Polymerase Chain Reaction (PCR) on serum;
- Detection of Circulating Anodic Antigen (CAA) on serum and/or urine;
- Anti-*Schistosoma* antibody seroconversion.

**Chronic schistosomiasis**

POSSIBLE

Signs and/or symptoms of urogenital schistosomiasis (e.g., haematuria, bladder mucosal thickening/masses, hydroureteronephrosis, bladder cancer, infertility).

OR

Signs and/or symptoms of hepato-intestinal schistosomiasis (e.g., chronic diarrhoea, colon pseudopolyps, liver fibrosis, portal hypertension).

In individuals who have lived in or travelled to endemic countries in a period of time that is at least 3 months before presentation; particularly if they report contact with freshwater.

PROBABLE

Positive anti-*Schistosoma* serology.

AND

In individuals who have lived in or travelled to endemic countries in a period of time that is at least 3 months before presentation; particularly if they report contact with freshwater.

PROVEN

At least one among the following:

- Eggs in stool, urine or tissue biopsy;
- Detection of *Schistosoma* DNA by PCR on serum, stool and/or urine;
- Detection of CAA on serum and/or urine.

## Recommendations for Katayama syndrome

### Diagnosis

#### When Katayama syndrome should be considered?

##### Recommendations

The diagnosis of acute schistosomiasis should be considered in subjects with the epidemiological criterion (travel in an endemic area in the last 3 months and history of direct contact with fresh water) and at least one among the following:

- Onset of symptoms such as fever, myalgia, non-productive cough, sweating, gastrointestinal symptoms, hepatomegaly, urticarial rash, neck pain.
- Eosinophilia

##### Evidence summary

The term acute schistosomiasis, includes two entities: cercarial dermatitis and Katayama syndrome.

Cercarial dermatitis consists of a maculopapular and itchy rash that affects skin areas exposed to fresh water contaminated by *Schistosoma* spp cercariae. It usually appears within 72 h from exposure and may persist for up to 15 days [13]. However, these guidelines are not intended to cover further details on cercarial dermatitis.

Katayama syndrome consists of a plethora of signs and symptoms that could develop several weeks or even months (usually 3–12 weeks) after infection [14]. It usually manifests in non-immune individuals, travellers or people living in endemic countries but without previous exposure, who are exposed to their first infection. Rarely, it may result from heavy reinfection or *S. japonicum* infection among chronically exposed populations [15]

Katayama syndrome is considered to be an immunological reaction due to the migrating and maturing larvae of *Schistosoma* spp. [14]. The hypothesis of a possible role of egg deposition in causing signs and symptoms is less convincing. Notably, a recent study has demonstrated that Katayama syndrome may be elicited in subjects infected by male parasite only, thus, without eggs production [16, 17].

Signs and symptoms usually present suddenly (Table 1). Most patients recover spontaneously in 2–10 weeks, while a small percentage may develop more serious disease and life-threatening complications have been rarely reported [18].

**Table 1:** Katayama syndrome clinical presentation

Common signs and symptoms	Fever, headache, myalgia, non-productive cough, sweating, urticarial rash, neck pain, abdominal tenderness (migration of juvenile worms) and patchy pulmonary infiltrates on chest radiograph [14, 20]
Severe presentation	Weight loss, diarrhoea, dyspnoea, severe abdominal pain, hepatomegaly and generalized rash
Life-threatening complications	Neurological, sporadic reports of cardiac involvement [21, 22]

Clinical presentation may slightly vary according to the parasite species, gastrointestinal symptoms being almost exclusive in *S. mansoni*, while urticarial rash being somewhat more common in *S. haematobium* infection [18]. Asymptomatic infection is common [14].

Eosinophilia is often present and serves as a diagnostic clue. There is no consensus about the best cut-off of eosinophil count to raise suspicion in travellers. The onset of eosinophilia is often delayed by several days as compared to clinical syndrome. In 25% of cases, eosinophils can be within the normal range [18, 19].

The non-specific presentation and the temporal delay between the exposure and the clinical onset explain why it is the imported form of schistosomiasis that is most likely to be misdiagnosed in non-endemic countries [14].

#### Which specific laboratory tests should be used in patients with clinical suspicion of Katayama syndrome?

##### Recommendations

It is recommended to use a combination of direct and indirect tests to diagnose Katayama syndrome:

- One serological test with high sensitivity and specificity. If serology is initially negative repeat the test after 3–4 weeks from the onset of symptoms and approximately 4–8 weeks after contact with contaminated water to check for seroconversion
- Parasitological examination of at least 3 stool/urine samples. If initially negative repeat the test after 4–8 weeks from the exposure.

In case of availability, you may consider using:

- PCR on serum;
- CAA detection on serum and/or urine.

##### Evidence summary

Parasitological confirmation of Katayama syndrome is challenging because at the onset of clinical signs both serology and eggs detection on stool and urine are often negative. It may be even harder in lightly infected travellers since they are likely to harbour a low worm burden. Consequently, these tests must often be repeated several times after the infection is suspected clinically.

Eggs production begins at the end of juvenile worms’ migration to venous plexuses when adult development is complete. The timeframe by which this occurs depends on the schistosome species but usually lasts 4–8 weeks after skin penetration of the larvae [18]. However, considering that the onset of symptoms can be delayed by more than 8 weeks, it is possible that eggs may already be detectable in stool or urine when patients present to medical observation [23].

Definite diagnosis relies on seroconversion of anti-*Schistosoma* antibodies. Serological tests are usually negative at the clinical onset and immunological investigations should be repeated 3–4 weeks from the onset of symptoms and approximately 4–8 weeks after contact with contaminated water to detect seroconversion [18, 24].

In addition, the sensitivity of immunological methods is low for tests that make use of egg antigens whereas assays based on worm antigen show a better performance [18, 25].

New tools such as PCR and antigen detection have been employed in the diagnosis of Katayama syndrome in the research setting and, at present, their role in routine diagnosis has to be determined.

Molecular methods have been shown to enable diagnosis in exposed travellers with the highest sensitivity, at an earlier stage than conventional tests and enabling species detection [26, 27]. Notably, parasitic DNA seems to be already present in the bloodstream during the prepatent phase and later in other specimens such as stool and urine [26].

Among the antigen detection methods, the up-converting phosphor-lateral flow (UCP-LF) CAA serum assay was demonstrated to be the most sensitive method for the diagnosis of acute schistosomiasis in a group of tourists with PCR-confirmed infection when compared both to adult worm antigen and soluble egg antigen based serological assay [28].

### Treatment and follow-up

#### How Katayama syndrome should be treated?

##### Recommendations

- All patients with probable or proved Katayama syndrome should be treated with an antiparasitic drug associated with steroids administration;
- Empiric treatment with PZQ may be considered before the parasitological confirmation in travellers from endemic countries who presents with possible acute schistosomiasis;
- PZQ dosage for acute schistosomiasis: 40 mg/kg/day for *S. mansoni* and *S. haematobium* and 60 mg/kg/ day for *S. japonicum,* both in 2 divided doses the same day for 1–3 days associated with steroids administration (prednisone 25 mg/die or equivalent for 3–6 days with progressive de-escalation during 2–3 weeks). Repeat PZQ in 6–8 weeks when all worms will have developed into adults.

##### Evidence summary

To date, there have been not randomized controlled trials studying treatment approach to manage Katayama syndrome among travellers returning from endemic countries. Moreover, treatment is often administered according to clinical suspicion, given that infection is usually confirmed retrospectively [14].

Considering that the pathogenesis Katayama syndrome seems primarily related to the immunological response to migrating juvenile parasites, on which PZQ is not effective [29], the administration of antiparasitic therapy alone has been largely questioned [14]. Moreover, anti-parasitic treatment alone can cause a worsening of clinical presentation due to an allergic-like reaction in around 50% of patients, possibly due to adult parasites lysis by PZQ [19, 30, 31].

For all those reasons, most authors suggest to associate steroids with PZQ to control the inflammatory phenomena [19, 32–34].

Accordingly, PZQ may be used either in combination with corticosteroids [35, 36], after corticosteroids [37, 38] or after mature worms have started to lay eggs (about 1–2 months from exposure) [18, 21, 34]. Notably, the use of steroids has been shown to decrease plasma levels of PZQ by 50% [39].

Whatever schedule is chosen, the antiparasitic treatment must be repeated when all schistosomulae and juvenile worms are supposed to have reached the adult stage to prevent chronic infection. This usually occurs 1–2 months after skin penetration[18] and, accordingly, PZQ should be repeated after 6–8 weeks from the first dose.

The recommended praziquantel dosage does not differ from that recommended for chronic schistosomiasis [14, 36]. No consensus exists about the antiparasitic treatment duration at the clinical presentation, with most authors suggesting to continue for 1–3 days when it is started[13, 14, 23, 36].

The use of artemether in acute schistosomiasis has been investigated because its addition holds the potential for increasing the cure rate because of its activity against young *S. japonicum* schistosomulae[40] and its prophylactic effect against *S. mansoni* [41]. However, studies have been set aside because of the risk of selective pressure on *Plasmodium* spp. if employed in control activities [41]. At present, there is no evidence supporting the use of arthemeter-praziquantel combination treatment routinely.

It is suggested to perform stool and urine microscopy after a complete treatment. Individuals with evidence of active infection (viable eggs) should be re-treated with a standard PZQ dose.

New diagnostic tools have been employed in the treatment response follow-up with promising results. While Wichmann et al. reported a significant reduction of PCR Ct (cycle threshold) values, with 50% of patients resulting negative after treatment among travellers with baseline PCR positive results [27], these results were not confirmed [28]. Indeed, in the latter study, the participants were followed both by PCR and UCP-LF CAA assay: while CAA in serum and urine became negative after treatment in almost all individuals, the majority remained PCR positive [28].

In conclusion, there is not sufficient evidence to recommend a specific follow-up for patients with acute schistosomiasis and further evidence has to be collected.

## Recommendations for chronic schistosomiasis

### Screening

#### Who should be screened for chronic schistosomiasis?

##### Recommendations

- Screening for chronic schistosomiasis is recommended in all subjects, included asymptomatic, who were born or have lived for at least 6 months in endemic countries;
- Screening is also recommended in all subjects, independent of their country of origin, who have visited endemic countries even for short periods (e.g., tourists) and who may not exclude freshwater exposure.

##### Evidence Summary

Recent national and international guidelines recommend screening for *Schistosoma* spp. infection in migrants from endemic countries (Table 2) irrespective of their symptoms or age [42–48].

**Table 2:** Schistosomiasis endemic countries

Africa		
Angola	Guinea	Democratic Republic of Congo
Benin	Guinea-Bissau	Rwanda
Botswana	Equatorial Guinea	Sao Tome e Principe
Burkina Faso	Kenya	Senegal
Burundi	Liberia	Sierra Leone
Cameroon	Libya*	Somalia
Côte d’Ivoire	Madagascar	South Africa
Chad	Malawi	Sudan
Congo	Mali	South Sudan
Egypt	Mauritania	Swaziland
Eritrea	Mozambique	Tanzania
Ethiopia	Namibia	Togo
Gabon	Niger	Uganda
Gambia	Nigeria	Zambia
Ghana	Central African Republic	Zimbabwe
Asia		
Saudi Arabia*	Indonesia*	Syria*
China*	Iraq*	Yemen
Cambodia	Laos	
Philippines	Oman*	
South America		
Brazil *	Suriname*	
Saint Lucia (Caribbean)*	Venezuela*	

*Different transmission status within the country

Interruption of transmission to be confirmed (screening in asymptomatic subjects usually not recommended): Algeria, Antigua and Barbuda, Djibouti, Japan, Jordan, Guadeloupe, India, Iran, Lebanon, Malaysia, Morocco, Martinique, Mauritius, Montserrat, Puerto Rico, Dominican Republic, Thailand, Tunisia, Turkey.

Additional countries and territories not previously considered endemic for schistosomiasis where transmission has been reported recently (screening in asymptomatic subjects usually not recommended): Myanmar, Nepal and Pakistan in Asia, Corsica (France) and Almeria (South-East Spain) in Mediterranean Europe [9, 10, 49, 57, 58]

Source: IAMAT, WHO.

According to WHO, the endemic areas for schistosomiasis are the following.

- Africa, mainly sub-Saharan and Egypt;
- East coast of Brazil, Venezuela, Suriname and some Caribbean islands;
- Middle East, some circumscribed areas of China and Indonesia, Laos, Cambodia, the Philippines

However, transmission has been reported in geographic areas not previously considered endemic: Myanmar, Nepal and Pakistan in Asia, Corsica (France) and Almeria (South-East Spain) in Mediterranean Europe [9, 10, 49–51].

International guidelines agree on screening for schistosomiasis as soon as migrants enter the destination country [42, 44, 45, 52]. On the other hand, as schistosomiasis is a chronic and progressive disease, offering screening tests only to recently arrived migrants might leave undiagnosed high-risk subjects who have lived in non -endemic countries for a long time or who have arrived illegally.

Canadian guidelines on migrants’ screening are among the few documents that recommend universal screening for all subjects who were born o have lived in endemic area irrespective of the date of arrival in the destination country [42].

The Centers for Disease Control and Prevention (CDC) recommend screening all subjects who have spent at least 6 months in rural areas of endemic countries and who have reported exposure to potentially contaminated freshwater [53]. The use of freshwater exposure history as an inclusion criterion to access screening may be misleading because of a possible recall bias, particularly among expatriates living in the tropics for long periods [54].

Screening for schistosomiasis is particularly important for individuals affected by chronic viral hepatitis (HBV and HCV). Indeed, portal fibrosis due to schistosomiasis may complicate the progression of HBV and HCV-related liver disease [55, 56].

#### How to screen for schistosomiasis?

##### Recommendations

Serological tests are the recommended screening tools considering their high sensitivity for the detection of schistosomiasis in low-endemicity settings.

##### Evidence Summary

Some of the European [44, 45, 48], Canadian [42] and Australian [43] guidelines recommend the use of serology to screen asymptomatic subjects at risk of schistosomiasis.

A different approach is supported by the CDC guidelines and indicates presumptive treatment with PZQ for SSA migrants while screening by serology is limited to subjects for whom antiparasitic treatment is contraindicated or in case of PZQ shortage [46].

Recently, a model study designed for the Canadian setting found the presumptive treatment to be less expensive and more effective than watchful waiting or screening if the prevalence of schistosomiasis in the target population was greater than 2.1% [59].

A similar economic evaluation conducted in the Italian setting was recently published [60].

In the Italian setting, the application of presumptive treatment is hindered by several factors. First, there is no formal migrant reception policy in Italy and most migrants arrive in Europe by illegal means, making a structured screening program not feasible. Second, PZQ is not licensed in Italy and written, informed consent is needed before a prescription to each patient. This procedure does not allow the application of such a strategy on a large scale. Third, PZQ availability is limited and only reference centres are able to receive a regular supply.

Screening by parasitological tests relies on the identification of *Schistosoma* spp. eggs in stool or urine, its sensitivity is low (under 50%) [61] and the collection of more than one sample in order to increase the sensitivity is challenging in the screening context.

A promising tool in the screening setting is the detection of the CAA antigen in serum and urine, which has demonstrated high sensitivity and specificity even in asymptomatic patients with chronic infection (87–95% sensitivity and 100% specificity) [62, 63]. Unfortunately, at present, the absence of a commercial kit hinders its wide use.

Therefore, as published in most guidelines, we recommend to perform serologic screening in eligible patients because of its high sensitivity and good specificity, even if cross reaction with other helminths may occur. The performances of the commercial assays are similar for *S. mansoni* e *S. haematobium* [61], while for the Asian species and for *S. intercalatum*, *S. guineensis* and hybrids, a reduced sensitivity is expected [25, 64]

ELISA and immunochromatographic (ICT) assays are widely available in non-endemic countries and have shown fair sensitivity and specificity [61, 65].

### Diagnosis

#### When chronic schistosomiasis should be suspected?

##### Recommendations

The diagnosis of chronic schistosomiasis should be considered in subjects who present the epidemiological criterion (travel or origin from an endemic area) and at least one among the followings:

- Signs and/or symptoms, even if non-specific, affecting the gastrointestinal system (abdominalgia, hepato- and/or splenomegaly);
- Signs and/or symptoms, even if non-specific, affecting the urogenital system (e.g., haematuria, dysuria, lower back pain, hemospermia);
- Eosinophilia.

##### Evidence summary

The onset of signs and symptoms of chronic schistosomiasis usually occurs weeks to years after infection because of the slow process of chronic inflammation that leads to the disease manifestations [66].

Hepato-intestinal schistosomiasis is generally paucisymptomatic, but can seldom cause mild abdominal discomfort with colicky pain and/or bloody mucous diarrhoea [66].

Chronic inflammation of the intestinal mucosa and the resulting fibrosis can lead to abdominal adhesions, calcifications, colonic polyps with consequent constipation and intestinal obstruction [66]. In 5–15 years, around 20% of infected patients develop a progressive pre-sinusoidal periportal fibrosis, developing from the inflammation around eggs transported to the liver through the portal system, that leads to portal hypertension, splenomegaly and varices [66].

Liver function is usually preserved until the late phase of the disease; at this stage ascites and decompensated liver disease may occur [66]. Liver fibrosis develops more frequently and more rapidly in *S. japonicum* and *S. mekongi* infection [24, 67] whereas severe cirrhosis and liver function abnormalities are more often caused by HBV or HCV co-infection [68]

*S. haematobium* adult worms reside in the pelvic venous plexus, and the symptoms and pathological modifications of urogenital schistosomiasis are closely related to the trapping of eggs in the bladder wall and genital organs [66].

Usually, the first signs of urogenital schistosomiasis are haematuria, dysuria, lumbar and perineal pain[69].

The bladder mucosa may present ulcers, thickenings, pseudopolyps, masses, scarring processes and calcification. Granuloma of the ureteral walls may cause hydronephrosis and chronic renal insufficiency in later stages [66].

The most worrisome consequence of urogenital schistosomiasis is bladder cancer [70]. For this reason, *S. haematobium* in classified among the biologic carcinogens by the International Agency for Research on Cancer (IARC) [71].

*S. haematobium* is also responsible for the genital disease that affects both females (female genital schistosomiasis or FGS) and males (male genital schistosomiasis or MGS) [72].

In the female of all ages, FGS may cause vulvo-vaginal changes such as hypertrophic or ulcerative lesions, fistulae and other pictures similar to condyloma plana. Moreover, FGS may cause cervicitis, salpingitis, chronic abdominal pain, vulvar pruritus, vaginal discharge, vaginal bleeding following sexual intercourse and menstrual disorders [73]. Sometime genital schistosomiasis may be misdiagnosed as sexually transmitted infections.

Characteristic lesions may be observed by colposcopy [74]. FGS has also shown to correlate with repeated miscarriages, low birth weight infants, childbirth complications, infertility and higher risk of acquiring HIV and other sexually transmitted diseases [75–77].

Men are also affected by genital schistosomiasis; granulomatous lesions and consequent fibrosis of the genital tract and local vessels can cause recurrent prostatitis, orchitis, hemospermia, erectile dysfunction, hydrocele, phimosis, oligo-azoospermia with secondary infertility [78–80].

Besides the epidemiological criterion, when the clinical picture is suggestive, it is advisable to investigate schistosomiasis among the differential diagnoses because, as reported by literature, cases have been described in areas previously considered as non-endemic (e.g., Myanmar, Nepal, Pakistan, Almeria in South-East Spain, Corsica in France) [9, 10, 49, 57, 58].

Eosinophilia is often present in different percentages according to the cut-off applied: 42% using the cut-off of 0.4 × 10^9^ cells/l [81] and 48–52% using the cut-off of 0.3 × 10^9^ cells/l [82, 83].

Although frequently associated with schistosomiasis, it is important to remember that an elevated eosinophil count may be due to other concomitant helminthiasis [82, 84] and, vice versa, that eosinophilia may be absent in a high proportion of infected patients.

In subjects with a possible, probable and established diagnosis of schistosomiasis we recommend screening for other epidemiologically related infections (see Box 3).

BOX 3 Recommendations for co-infection screening The following screening tests are recommended in the subject with a possible, probable and established diagnosis of schistosomiasis to rule out other epidemiologically related infections for which screening is recommended by national and international guidelines [44, 45]:

- HIV-Ab;
- HCV-Ab, HbsAg, HBs Ab, HBc Ab, HAV-IgG (particularly recommended in subject with hepato-intestinal schistosomiasis);
- Strongyloides antibody.

Patients potentially eligible for latent tuberculosis infection (LTBI) treatment according to local guidelines may benefit from specific screening when signs or symptoms of active tuberculosis disease are excluded. IGRA (Interferon-Gamma Release Assays) or Mantoux test may be used.

## Which specific laboratory test should be used in patients with clinical suspicion of chronic schistosomiasis?

### Recommendations

A combination of direct and indirect tests to diagnose chronic schistosomiasis is recommended:

- At least one serological test with high sensitivity and specificity
- Parasitological examination of at least 3 stool/urine samples

In case of availability, the following additional tests may be considered:

- DNA detection tests on stool, urine and serum;
- CAA detection on serum and/or urine

Tissue biopsy may be considered in selected cases of high diagnostic suspicion when the diagnosis may not be confirmed by less invasive methods.

#### Evidence summary

Direct methods rely on the visualization of parasite eggs by optical microscopy [85] in different samples, traditionally stool and urine, but also in semen, biopsies of bladder wall, liver, intestine, and lower female genital tract [66].

When performed by experienced personnel, microscopy-based methods show a specificity of 100% while sensitivity varies with the intensity of infection, concentration technique and number of samples examined and it is frequently less than 50%, especially in patients with a low burden of infection [61, 85]. Eggs are excreted irregularly, therefore, the collection of at least 3 samples of urine and stool over different days increases the sensitivity of microscopy [86]. Recommendations for the collection of traditional biological samples are summarized in Box 4 [87].

A study from Côte d’Ivoire has shown that the traditional practice of asking patients to exercise before collecting urine samples does not improve the test sensitivity of microscopy [88]. The WHO no longer suggests this practice.

Eggs of *S. mansoni, S. japonicum, S. mekongi, S. intercalatum* and *S. guineensis* are generally identified in stool and *S. haematobium* in urine.

Eggs morphology, size and colour as well as spine position and size are specific characteristics of each *Schistosoma* species, although atypical morphology may occur.

A second type of direct diagnostic tool is represented by monoclonal antibody-based antigen detection techniques.

These antigens, namely Circulating Cathodic Antigen (CCA) and CAA, are produced in the worm intestine and released by living worms. As a consequence, their presence represents a sign of active infection and the quantity detected has been found proportional to the worm burden [89]. Therefore, antigens detection tools are suitable both for diagnosis and treatment response follow up [28, 63].

To our knowledge, no commercial test approved for clinical diagnosis by detection of CCA are currently available in Italy. Its sensitivity, compared to microscopy, varies broadly depending on the prevalence and intensity of infection and it is higher for *S. mansoni* (72–89%) with respect to *S. haematobium* (24–39%) [28, 61, 90]. Compared to specificity declared by the manufacturer (95%), other studies have shown much lower values, around 74–81% [91].

CAA detection on serum and urine is possible thanks to the UCP-LF technology developed by Leiden University Medical Centre in the Netherlands [92].

Its use in endemic countries has shown good performance in the diagnosis of infection caused by all species of *Schistosoma* spp. even in populations with low prevalence and low intensity of infection [93, 94].

In a retrospective study conducted in a non-endemic setting, CAA concentrations were determined in 81 serology-positive individuals and, based on detectable CAA levels, an active infection was diagnosed in 69% of the subjects, with migrants showing a higher level of CAA than travellers[95]. Another study conducted in the same setting compared the diagnostic performance of UCP-LF-CAA and the performance of all the available direct and indirect diagnostic tools versus a *Composite Reference Standard*, (CRS) among asymptomatic Eritrean refugees. UCP-LF-CAA on serum showed the highest sensitivity [62]. In another study carried out in a non-endemic setting, out of 23 patients with confirmed chronic schistosomiasis, 87% presented a CAA positive result, which was also positive in 20% serology-positive only (probable) schistosomiasis cases [63].

Considering these promising results, a commercial test would be most welcome.

It is possible to detect *Schistosoma* DNA in specimens potentially containing eggs or worms thanks to real-time PCR, loop-mediated isothermal amplification (LAMP) and recombinase polymerase amplification (RPA) [96].

Considering that cell-free DNA can be released by the parasite in various stages (e.g., by the living worm or by the degradation of the eggs) and may also be the result of dying or dead parasites, the test interpretation in not always univocal [97].

Most authors claim a specificity of virtually 100% for DNA detection tools, but sensitivity remains around 50% [62]. Of interest, these tools are able to identify the *Schistosoma* species in case of negative microscopy [97].

The absence of standard procedures and of robust data on test performance in large populations, the high specialization needed and the costs are at present the main limitations to the use of those diagnostic tools.

Serology is usually employed to determine whether or not a subject has been previously exposed to *Schistosoma* spp. infection. Seroconversion generally occurs within 4–8 weeks of infection, but the interval can be as long as 22 weeks [98].

The sensitivity of seroassays for schistosomiasis diagnosis is higher than that of microscopy and those tests have been successfully used for detecting infections in non-endemic areas, where patients have low egg burden [99]. Several commercial assays are available and the most used are based on four technologies: immune-chromatography test (ICT), ELISA, Western Blot and indirect hemagglutination test (IHT).

The majority of the antibody detection tests used in routine are based on *S. mansoni* antigens and, therefore, the sensitivity for *S. haematobium* and other species diagnosis may be sub-optimal [25].

In conclusion, direct tests are less sensitive than serologic assays. Nevertheless, microscopy, antigens and DNA detection tests, thanks to their high specificity are useful to confirm the diagnosis of active schistosomiasis.

Therefore, it is suggested to perform several tests and to define the diagnosis as confirmed when at least one direct test is positive.

BOX 4. Recommendations for collection and storage of biological samples **STOOL**

- It is advisable to analyse 3 different stool specimens collected every other day, at any time during the day;
- Stool should be collected on a dry surface, and should not be contaminated by urine or water;
- On average,10 g from different stool portions should be transferred to a screw-cap plastic container;
- For parasitological diagnosis, it is possible to store stools using fixatives such as formalin 10%, sodium acetate-acetic acid-formalin (SAF)

or ecological alternatives often available in OneVial stool collection systems;

- For molecular diagnosis, it is suggested to extract DNA from fresh stools;
- The sensitivity of parasitological diagnosis is increased by using concentration methods such as Ridley’s sedimentation technique or flotation kits.

**Urine**

- Suggest the patient to collect at least 10 ml of the terminal urine for three consecutive days and preferably between 10 am and 2 pm when the circadian excretion of eggs is higher;
- Alternatively, a 24-h urine collection of terminal urine can be made;
- Protection of the specimens from the light is suggested during the transportation to the laboratory to avoid hatching if the viability of eggs is to be evaluated;
- It is no longer recommended to collect urine samples after physical exercise;
- It is preferable to analyse samples as soon as possible after the collection, at maximum within 24 h of their collection

## Which radiological imaging should be performed in patients with chronic schistosomiasis?

### Recommendations

#### Recommendations

Ultrasound (US) of the abdomen is recommended in the following situations:

- Symptoms suggestive of chronic schistosomiasis;
- Signs suggestive of chronic schistosomiasis (e.g., haematuria, microhaematuria, splenomegaly, history of hematemesis);
- Comorbidities (e.g., HBV or HCV infection);
- Positive parasitological examination of stool or urine or other direct diagnostic methods such as CAA or DNA detection tests.

US in asymptomatic patients affected by chronic schistosomiasis with negative parasitological tests (isolated positivity of serology) may still be offered if economic and organizational resources are available.

##### Evidence summary

According to recent reviews, abdominal US is the most widely used imaging method for the initial evaluation of both hepato-intestinal and urinary chronic schistosomiasis [100].

The prevalence of US-identifiable lesions seems markedly different among patients with a positive parasitological test compared with asymptomatic patients with only positive serology (34.9% [82] vs 2.89% [101], respectively)[101].

In urogenital schistosomiasis, bladder wall alterations (thickening, pseudopolyps, luminal masses), post-voiding residue, ureteral or bladder stones and hydroureteronephrosis are the most commonly described lesions[102]. US represents a valid option in the evaluation of bladder cancer in order to avoid invasive diagnostic methods. For example, *Santos* et al. carried out a study aimed at comparing US and cystoscopy (the latter used as *gold standard*) in 80 *S. haematobium* infected patients with bladder lesions. US showed 100% sensitivity and 72.9% specificity in detecting neoplasms later confirmed by cystoscopy [103].

In patients with hepato-intestinal schistosomiasis, the most common US findings are peri-portal fibrosis, increased portal vein diameter, splenomegaly and gallbladder wall thickening [104].

Periportal fibrosis is classified according to the Niamey Belo Horizonte imaging pattern classification [104].

US findings are useful to estimate the risk of complications. In particular, the “*Schistosoma mansoni* score” (SMS), calculated on the basis of periportal fibrosis patten and the diameter of the portal vein divided by the individual’s height, correlates with the presence and severity of oesophageal varices: score ≥ 2 is 95% sensitive and 58% specific for the presence of varices; score ≥ 3 is strongly correlated with risk of variceal bleeding [105]. On the contrary, the role of portal and splenic vein doppler US has not been established by clinical studies yet [106], as well as that of liver and spleen stiffness.

#### When a cystoscopy should be performed in patients with urogenital schistosomiasis?

##### Recommendations

- Cystoscopy ± biopsy is recommended in patients with urogenital schistosomiasis if signs such as haematuria or bladder lesions persist > 6 months after PZQ therapy;
- Cystoscopy with biopsy can be considered in case of bladder imaging highly suggestive of neoplasia that does not begin to regress after 2–3 months after PZQ treatment;
- Cystoscopy ± biopsy is not recommended in the initial work-up of patients with a clinical suspicion of urogenital schistosomiasis since the diagnosis can be obtained through non-invasive tests (such as serology and parasitological test).

##### Evidence summary

Cystoscopy with biopsy is often used as a diagnostic tool for urogenital schistosomiasis, even if, as mentioned above, the diagnosis can be obtained with non-invasive tests (e.g., urine parasitological test, serologic tests, US) [83, 107]. A systematic revision identified 29 case reports and case series in which bladder biopsies were performed in lesions that subsequently resulted to be related to schistosomiasis and that responded to PZQ treatment [108].

The dynamic of bladder lesions after treatment suggests that the lesions usually begin to regress very quickly after PZQ treatment and, therefore, it is reasonable to perform cystoscopy with biopsy only in patients with signs (e.g., haematuria) or bladder lesion which persist > 6 months after treatment [109, 110]

Moreover, cystoscopy with biopsy is recommended in the evaluation of bladder lesions that have features suspected of malignancy and do not regress with antiparasitic therapy after 2–3 months and [83, 109, 110].

#### When a colposcopy should be performed in patients with suspected genital schistosomiasis?

##### Recommendations

- Colposcopy is recommended in women suspected of FGS if signs and symptoms of genital involvement are present to verify differential diagnoses and co-morbidity.
- Colposcopy is recommended in women who complain of genital discomfort even before the diagnosis of chronic schistosomiasis is formulated. A serology test is recommended in case of a suggestive clinical picture.

##### Evidence summary

FGS manifests with unspecific symptoms [111]. Colposcopy is the main diagnostic tool to detect cervical and vaginal lesions of FGS that may occur in various combination: grainy sandy patches, homogeneous yellow sandy patches, abnormal vascularization and rubbery papules being most common [112].

Whereas clinical findings detected by visual inspection may serve as an adequate diagnosis for FGS in *S. haematobium* endemic areas [111], in non-endemic countries the presence of colposcopic lesions consistent with FGS and a positive serology can serve as a diagnostic tool without performing biopsies. Indeed, in genital lesions, the eggs are located in highly focal clusters and could be missed during histological analysis [111, 113].

#### Other diagnostic tools in patients with chronic schistosomiasis

##### Computerized urotomography (uro-CT)

Uro-CT is helpful to identify ureteral dilatation, stenosis, and calcifications. Axial scans might document the presence of circular calcifications at the level of the ureteral wall, giving the “foetal head in pelvis” sign, as well pathognomonic. The presence of contrast-enhanced sessile masses in the bladder wall might suggest the presence of a neoplasm [114].

##### Rectal snip

Rectal snip consists of biopsies of rectal mucosa (at 8 cm from the anal canal) performed during rectoscopy to identify *Schistosoma* spp. eggs. In a clinical study conducted on a cohort of expatriates, its sensitivity was 61%, while stool examination sensitivity was only 39%[115]. Rectal snip is nowadays rarely used because of its invasiveness.

### Treatment

#### Who should receive antiparasitic treatment for chronic schistosomiasis?

##### Recommendations

- All patients with probable or proved chronic schistosomiasis must receive specific antiparasitic treatment (PZQ)
- Empiric antiparasitic treatment with PZQ may be considered in i) migrants from countries with a high prevalence of schistosomiasis in the context of public health initiatives; ii) patients with high clinical suspicion of schistosomiasis but without a parasitological confirmation.

### Evidence summary

Available first-line treatment is able to kill adult worms and consequently block eggs deposition. If untreated, schistosomiasis can persist for years and chronic infection can lead to an increased risk of liver fibrosis or bladder cancer[53].

Antiparasitic treatment is recommended even in cases of probable diagnosis because of the variable performance of the tools available to confirm schistosomiasis diagnosis and the risks associated with delayed treatment.

This approach is possible thanks to PZQ efficacy and good safety profile[72, 116].

While presumptive treatment in asymptomatic subjects can prevent the onset of advanced schistosomiasis, when a subject is diagnosed following the onset of clinically evident schistosomiasis, treatment does not guarantee complete regression of the organ damage caused by fibrosis or cellular transformation [24, 59].

#### What is the recommended antiparasitic treatment for schistosomiasis?

##### Recommendations

- PZQ is the first line antiparasitic treatment for schistosomiasis;
- PZQ dosage for chronic schistosomiasis: 40 mg/kg/day po in 1 or 2 divided doses the same day for *S. haematobium* e *S. mansoni*, 60 mg/kg/day in

3 divided doses the same day for *S. japonicum* e *S. mekongi*;

- Calculate the final dose on the patient’s weight and administer divided doses 4–6 h apart. It is recommended to take the drug with or immediately after a meal to guarantee optimal absorption;
- In non-endemic areas, it is suggested to repeat the daily dosage for three consecutive days for non-pregnant and non-breastfeeding subjects ≥ 4 years old with chronic schistosomiasis.

Investigate the past history of seizures or skin nodules and clinically rule out neurocysticercosis or ocular cysticercosis before prescribing PZQ.

##### Evidence summary

PZQ remains the drug of choice for the treatment of schistosomiasis [24, 117].

It is an acylated quinoline-pyrazine with activity against all schistosome species and it is mostly marketed in oral formulations (600 mg tablets). The dosage and the number of daily doses are individualized based on the patient’s weight and are summarized in Table 3. PZQ can be administered as a single dose or divided into two doses 4–6 h apart, although studies have not shown a significant difference in the risk of developing adverse events [118, 119]. It is recommended to take it with or immediately after a meal to enhance its absorption [120]. Dosage must not be adjusted in case of chronic kidney disease or mild liver insufficiency, but caution should be taken in patients with liver failure (Child–Pugh Class B or C), because of the risk of higher plasma concentrations of unmetabolized product [121].

**Table 3:** Recommended PZQ dosage depending on the aetiological agent

	Dosage recommended by the WHO^a^	Cure rate of the single-day treatment^b^
*S. haematobium*	40 mg/kg/die in one or two doses (4–6 h apart) on the same day	77.1% (95% CI 68.4–85.1%)
*S. mansoni*	40 mg/kg/die in one or two doses (4–6 h apart) on the same day	76.7% (95% CI 71.9–81.2%)
*S. japonicum* *S. mekongi*	60 mg/kg/die in one or two doses (4–6 h apart) on the same day	*S. japonicum*: 94.7% (95% CI 92–98%)

^a^The current consensus document suggests to repeat the daily PZQ dosage for three consecutive days in non-endemic setting for non-pregnant and non-breastfeeding subjects ≥ 4 years old with chronic schistosomiasis

^b^Cure rates derived from a metanalysis of studies conducted in endemic areas. The cure rate was calculated after one administration of a single-day treatment of PZQ [122]

PZQ is known to be efficient solely on adult parasites, having little or no effect on eggs and immature worms [117]. This is particularly relevant in the setting of acute schistosomiasis.

Studies carried out in SSA report an average 75% cure rate and 85-95% egg reduction rate after a single intake of PZQ [122, 123]. These data are acceptable in endemic settings where it is expected that patients are continuously exposed to risk of reinfection and the principal aim of the treatment is to reduce the worm burden [124]. By contrast, in non-endemic settings the objective of the treatment is to eradicate the parasitic infection in the individual patient considering a little or no risk of reinfection [125, 126].

The usefulness of a repeated dosage of PZQ has been investigated in high-risk communities in Africa: improvements in parasitological cure rates were, for *S. mansoni*, 69–91% after two doses vs 42–79% after one dose and, for *S. haematobium*, 46–99% after two doses vs 37–93%. The interval between the two administrations varied between 2 and 8 weeks [127]. In addition, a randomized clinical trial conducted in Côte d’Ivoire compared the cure rate at 10 weeks in patients receiving either a single dose or four repeated doses every two weeks. The cure rate at 10 weeks by parasitological examination was 42% in the first group vs 86% in the latter [128]. However, the above studies were conducted in an endemic area and authors could not exclude that the difference could be due to reinfection during the study period.

Again, there is still no consensus regarding re-administrations of PZQ in chronic schistosomiasis and an extreme variability in regimens has been shown in studies conducted in non-endemic areas [118].

CDC guidelines suggest a second dose after 2-4 weeks especially in lightly infected patients, as the immune response may be less robust in these subjects [53] On the other hand, in some European countries (e.g., Italy and Germany) it is common to provide three doses of PZQ standard dosage over three subsequent days to offer more than one dose to an extreme mobile population who are rarely able to attend a follow-up visit after 2-4 weeks [83, 125, 129].

### Pre-treatment evaluation

Before praziquantel intake it is mandatory to clinically exclude a concomitant neurocysticercosis. Indeed, praziquantel is effective against *Taenia. solium/Cysticercus cellulosae* and may cause an acute inflammatory reaction, which can lead to central nervous system (CNS) complications (seizures and stroke) and ocular damage. It is therefore recommended to look for any history of epilepsy, neurological signs and/or the presence of subcutaneous nodules[130].

In these subjects, a complete assessment including a CNS imaging and an ocular fundoscopic examination should be performed before administering the drug and seeking expert advice should be sought in case of suspected neurocysticercosis.

Moreover, interactions between antiretroviral drugs and PZQ must be taken in account when treating HIV positive patients and individuals with tuberculosis disease or LTBI [121, 131]

#### How PZQ should be used in special populations (pregnant and breast-feeding women, children, subjects with HIV, TB and LTBI)?

##### Recommendations

- Pregnancy and breast-feeding: PZQ may be used during pregnancy and breast feeding with a standard dosage (over 1 day). In non-endemic settings, the benefit of treating a pregnant woman must always be balanced with the risk of disease progression in the absence of adequate treatment and the possibility of losing at follow-up the woman.
- Children aged less than 4 years old: PZQ is recommended at standard dosage (over 1 day, off-label use of PZQ). It is possible to crush the tablets and administer them together with a soft food or drink.
- HIV co-infection: evaluation of drug interactions required.
- Patient with active TB or LTBI: administer PZQ prior to initiation of rifampicin therapy to avoid sub-optimal treatment for schistosomiasis.

### Evidence Summary

#### Pregnancy and breastfeeding

PZQ is listed among the “Class B” drug according to FDA classification. Retrospective studies and two double-blind randomized trials reported that there was no significant difference in the outcome of pregnancies in women who took the antiparasitic drug and the WHO declared that the benefits of taking PZQ during pregnancy outweigh the risks, therefore it encourages its use, both for targeted treatment and for control programs, during any trimester of pregnancy [132]. Although PZQ is found in small concentrations in breastmilk, WHO recommends its administration also during breastfeeding [132].

In non-endemic areas, the benefit of treating a pregnant, or breastfeeding, woman must always be balanced with the risk of disease progression and the possibility of losing her at follow-up [133].

#### Paediatric population

The safety of PZQ in children younger than 4 years has not been established. However, this population was treated during mass treatment campaigns and clinical studies, and no adverse events were reported. A phase II randomized trial conducted in 2017 demonstrated good tolerability of PZQ in children between 2 and 5 years old even if the efficacy was slightly lower in this age population compared to older children [134].

Thanks to those data on PZQ safety, in 2022 WHO guideline on control and elimination of human schistosomiasis preventive chemotherapy is mentioned as appropriate for preschool-aged children aged ≥ 2 years, while for those < 2 years, preventive chemotherapy may be considered for treatment on an individual clinical basis [124].

Nevertheless, without any other valid alternative, its prescription is still recommended at adult standard dosage.

In non-endemic areas, the risk–benefit ratio of treatment must be evaluated depending on the clinical manifestations and the risk of disease progression without adequate treatment.

If the child is not able to swallow a whole pill, WHO reports that it is possible to crush it and mix it with soft food or drink [124]. Nonetheless, a study by Coulibaly et al. raises suspicion that tablet crushing may alter the bioavailability and pharmacokinetic properties of PZQ [134].

A syrup formulation (Epiquantel^®^) exists but a single study has shown a lower cure rate with respect to standard formulation [135].

At present, an orodispersible formulation (arpraziquantel) is under the European Medicines Agency review. A phase II clinical trial aimed at evaluating its efficacy and safety in children between 3 months and 6 years showed promising results [136].

#### How patients with complicated chronic schistosomiasis should be managed?

##### Recommendations

After PZQ treatment, subjects with urogenital or hepato-intestinal schistosomiasis with organ complications should be managed with an individualized and multidisciplinary approach, possibly in a referral centre.

### Evidence summary

#### Hepato-intestinal schistosomiasis

Local granulomatous inflammatory response can lead to pre-sinusoidal inflammation and periportal fibrosis over the years [24, 137].

Four to eight percent of patients develop progressive portal hypertension, splenomegaly, hypersplenism and consequent risk of bleeding from rupture of oesophageal varices [138].

Non-selective beta-blockers, along with endoscopic ligature, are the first-line therapy in the prevention of esophageal varices bleeding. Pharmacological therapy alone can reduce to 2% the incidence of rebleeding at 2 years (vs 20% in placebo) [139].

First-line endoscopic treatments includes sclerotherapy and banding [137]. Variceal banding can reduce the frequency of rebleeding to 10% (vs 40% in pharmacological treatment alone) [137]. Transjugular intrahepatic portosystemic shunt (TIPS) is another minimally invasive procedure used in the management of portal hypertension and its complication after accurate patient selection [140].

Surgical techniques include non-derivative surgery (esophagogastric devascularization with splenectomy, called EGDS/azygo-portal disconnection and splenectomy, called APDS), and shunt procedures such as distal splenorenal shunt (DSRS) and Warren’s shunt [140].

A review published in 2018 did not find significant differences in a number of re-bleedings, adverse events or deaths between selective/non-selective shunt or devascularization procedure [138].

Splenectomy is, at present, the most studied and preferred intervention for the treatment of portal hypertension caused by schistosomiasis [137]. Nevertheless, it is accompanied by high invasiveness, non-negligible intraoperative mortality, risk of severe infection with capsulated bacteria and the consequences of post-splenectomy syndrome.

It is recommended to consider the risk of developing severe malaria in patients after splenectomy, especially for subjects coming from endemic areas for *P. falciparum* and *P. vivax* [141]. If inevitable, it is fundamental to accurately inform the patient about the importance of malaria prophylaxis and protection rules against mosquito bites.

### Urogenital schistosomiasis

The main complications of urogenital schistosomiasis are obstructive uropathy and bladder carcinoma [24].

Bladder cancer associated with *S. haematobium* infection are usually well differentiated and spread locally. Inflammation elicited by parasite eggs seems to act together with genetic predisposition as possible carcinogenic factors[71] and might increase the exposition of bladder epithelium to mutagenic factors originating from tobacco and chemicals [142].

In the case of low-grade superficial urothelial cancers (Ta, Tis, T1), transurethral resection of bladder tumor (TURBT) is usually performed [143]. Adjuvant intravesical chemotherapy instillation might be performed depending upon the risk group[143].

Immunotherapy (Calmette-Guérin bacillus and hemocyanin) seems to be superior to adjuvant chemotherapy in reducing recurrence in intermediate, high-risk cancer [143, 144]. This approach, widely used in non schistosomiasis-related bladder cancers, may not be equally effective considering the possible presence of other sites where the mucosa contains eggs or is already precancerous [143–145].

Radical cystectomy is the most frequently used intervention in low-grade carcinomas (T1-T2) whereas chemotherapy (gemcitabine and carboplatin) is reserved to advanced cancers (T3, T4) [146].

Regarding obstructive uropathy, it is most often caused by stenosing lesions of the ureters. It usually responds well to PZQ alone in children and adolescents. Adults with mild hydronephrosis can be treated with laser endoureterotomy, stenting or a combination of both[147]. In case of severe hydronephrosis, ureteral reimplantation is suggested [148].

### Follow up

#### How patients with chronic schistosomiasis should be followed-up after antiparasitic treatment?

##### Recommendations

- In asymptomatic subjects with probable infection in which only serologic data support the diagnosis, no follow-up is recommended.
- In subjects with eggs of *Schistosoma* spp. in stool or urine, parasitological monitoring is recommended 2–3 months after treatment with PZQ and, if viable eggs are still detected, a new antiparasitic treatment is recommended. Assessment of eggs viability is important since not viable eggs can be eliminated with excreta over time after parasitological cure.

However, if viability cannot be assessed or compliance with further parasitological follow-up (at 6 months) is uncertain, it is recommended to repeat praziquantel treatment in case of persistent eggs detection.

- In subjects with hepato-intestinal schistosomiasis and pathological findings at imaging, US monitoring is recommended 6 months after the end of treatment. The frequency of US follow-up may be modified depending on the severity of the picture and the presence of varices.
- In subjects with urogenital schistosomiasis with bladder wall lesions, US monitoring is recommended at 1, 3 and 6 months until lesions disappearance. The persistence of bladder lesions at 6 months after treatment should lead to histological investigation through biopsy to rule out carcinomatous evolution and differential diagnoses.

##### Evidence summary

Assessment of treatment response in chronic infection is a challenging issue in the management of schistosomiasis and relies on several tools.

Whole blood count is a cheap test and allows one to check the dynamic of eosinophils count.

In a study has been observed that, both in subject with a proved and probable diagnosis who presented eosinophilia at diagnosis, eosinophil counts normalized in all subjects at 12 months from treatment [63].

Normalization of eosinophil count was observed regardless of infection status or presence of other concomitant parasitosis at diagnosis. For this reason, even if it is useful to monitor the eosinophil count 6 months after treatment, its normalization alone cannot be used to define parasitological cure.

In the case of persistent eosinophilia, ruling out other helminthiases and other causes of eosinophilia, namely oncologic and autoimmune diseases, is mandatory [149].

Stool and urine microscopy is still the reference standard in the diagnostic process, despite low sensitivity, but it is even less sensitive when used in follow-up [150].

Considering the specificity and the low cost of microscopy, it is recommended to perform a control test (at least 3 samples of urine or stool) 2–3 months after treatment completion, including the assessment of eggs viability where feasible [52, 63]. Viability is assessed by microscopic observation of schistosoma eggs but non-reference laboratories could not be able to perform this evaluation routinely. In those cases, considering that most patients belong to a highly mobile population often characterized by poor compliance to follow-up, it can be recommended to retreat patients with persistent eggs excretion at the first control (2–3 months). This approach is also recommended by Australian guidelines [43].

This approach risks slightly increase the retreatment of individuals that are excreting non-viable eggs at 2–3 months. Therefore, if the physician is confident of the patient’s good compliance, it might be suggested to repeat a second microscopic control at 6 months after PZQ and repeat treatment if eggs are still present at this timepoint.

It is not strictly recommended to extend microscopic follow-up to 12 months. Indeed, some authors demonstrate that 12 months after treatment all patients with positive microscopy at baseline have cleared urine and stool [63, 91] and follow-up at 1 year in a generally healthy and highly mobile population is often challenging.

Of note, a negative parasitological test or the presence of non-viable eggs do not allow for confirming parasitological cure because of low sensitivity.

Serological tests are not useful as follow-up tools. Most patients may have positive serological results for more than 2 years after cure [151–153] even if among travellers, a quicker negativization of serology has been observed [153].

Furthermore, ELISA-based serology shows cross-reactions with other parasitic infection and consequently, false positive results must be considered [86].

Similarly, antibody-based antigen detection techniques performance on follow-up have been investigated. In a study conducted by Neumayr et al., CCA testing was performed 14 months after treatment and its specificity in follow-up was around 60–73% [91]. In another study, CCA testing demonstrated a higher specificity (92%) when interpreted by a semiquantitatively 4-point scale based on a 4-points scale defined by the intensity of the result line [62]. However, the lack of a unique system of interpretation hinders its use in follow-up.

On the other hand, the performance of CAA detecting tools in the evaluation of treatment efficacy seems to be promising both in acute and chronic schistosomiasis.

Indeed, being the CAA measured proportional to the infection intensity, in terms of the burden of adult worms [154] it would be the most suitable tool to detect the efficacy of treatment. In the study by Tamarozzi et al., the CAA concentration in serum, measured 6 months after treatment, decreased significantly from baseline in all CAA-positive patients at baseline [63]. A similar result was published by Hoekstra et al. where significant CAA levels were tested in urine 12–18 months after treatment of asymptomatic migrants [62]. In these studies, follow-up time points were rather delayed compared to treatment; however, importantly, CAA levels have been reported to drop early after treatment (6–8 weeks) [154, 155].

These results are particularly promising when dealing with migrants that are usually a dynamic and mobile population with poor compliance to follow-up. Considering these promising results, a commercial test would be most welcome.

Finally, DNA detection methods have been also tested to assess treatment efficacy.

Unfortunately, several studies reported DNA persistency, mainly in serum; whether this is due to the continuous release of parasite DNA from trapped eggs, dead or dying worms or because of surviving adult worms remains to be determined [28, 62, 85, 156].

Follow-up includes imaging monitoring in patients with portal hypertension, fibrosis, splenomegaly and bladder lesions.

Whereas initial hepatic fibrosis and splenomegaly can regress after PZQ therapy in young patients, among adults advanced alterations do not improve even after repeated therapeutic courses [100, 157]. The evolution to advanced stage of the disease is strictly dependent on complications’ onset and related treatment and no single follow-up scheme suitable for all patients can be recommended [137].

By contrast, US monitoring is an essential tool to follow up patients with urinary schistosomiasis after treatment, with most studies showing a regression of bladder lesions after 3–6 months [110, 158]. In a retrospective Italian study, 40 migrants with known urinary tract lesions were followed up with ultrasound performed on average 4 months after PZQ administration and 50% of patients experienced a complete regression of the lesions [83]. In a prospective study, Tamarozzi et al., observed the disappearance of bladder lesions at 6 months US follow-up in 16 followed-up patients [110]. A slow regression of upper urinary tract dilatation is possible [104].

Observing evident reduction in number and size of lesions at 1 and 3 months should probably reassure of the likely positive outcome and anticipate, with a good estimate, ultrasound findings at the 6-month follow-up [110]. This could be particularly relevant in young subjects who usually cannot be followed up until the complete resolution of lesions because of their migration plan.

Importantly, if a new exposure occurs after treatment, the recurrence of a new bladder lesion within 12–24 months should be more probably to ascribe to a new infection rather than to a PZQ treatment failure [104, 159].

Carcinogenic evolution [160] or other infections with a similar presentation, notably renal tuberculosis, must be ruled out if lesions or signs such as haematuria are persistent 6 months after treatment or also only slightly reduced at 3 months. In these cases, it is necessary to evaluate the persistence of active infection (e.g., presence of viable eggs) and evaluate adherence to the PZQ and, only when these options have been excluded, is cystoscopy with biopsy recommended [161].

## Conclusions

While considering the limitations due to the use of expert opinion-based method to rise consensus and the lack of broadly accepted definitions of acute and chronic schistosomiasis, these recommendations aim to help clinicians and healthcare workers to identify and manage schistosomiasis in the non-endemic setting. Considering some peculiarities of the Italian context, these recommendations may not be suitable in other non-endemic countries.

### Supplementary Information

Below is the link to the electronic supplementary material.

Supplementary file1 (DOCX 21 KB)

## Data Availability

Non applicable.
