# Supplementary material for: Schistosomiasis in non-endemic areas: Italian consensus recommendations for screening, diagnosis and management by the Italian Society of Tropical Medicine and Global Health (SIMET), endorsed by the Committee for the Study of Parasitology of the Italian Association of Clinical Microbiologists (CoSP-AMCLI), the Italian Society of Parasitology (SoIPa), the Italian Society of Gastroenterology and Digestive Endoscopy (SIGE), the Italian Society of Gynaecology and Obstetrics (SIGO), the Italian Society of Colposcopy and Cervico-Vaginal Pathology (SICPCV), the Italian Society of General Medicine and Primary Care (SIMG), the Italian Society of Infectious and Tropical Diseases (SIMIT), the Italian Society of Pediatrics (SIP), the Italian Society of Paediatric Infectious Diseases (SITIP), the Italian Society of Urology (SIU)
Source: Infection. 2023 Jul 7;51(5):1249–71. doi: 10.1007/s15010-023-02050-7 (PMC10545632; doi:10.1007/s15010-023-02050-7)
Supplement: Supplementary file 1 — Supplementary file1 (DOCX 21 KB) [file 15010_2023_2050_MOESM1_ESM.docx]

Supplementary Table 1: results of Delphi consensus-seeking procedure

| **ACUTE SCHISTOSOMIASIS** | | | | | | | | | |
| --- | --- | --- | --- | --- | --- | --- | --- | --- | --- |
| Statement | LIKERT SCALE | | | | | | | | |
|  | 1 | 2 | 3 | 4 | 5 | 6 | 7 | 8 | 9 |
| **When acute schistosomiasis (Katayama syndrome) should be considered?**  The diagnosis of acute schistosomiasis should be considered in subjects with the epidemiological criterion (travel in endemic area in the last 3 months and history of direct contact with fresh water) and at least one among the following:  a) onset of symptoms such as nocturnal fever, myalgia, non-productive cough, sweating, gastro-intestinal symptoms, hepatomegaly, urticarial rash, neck pain.  b) eosinophilia |  |  |  |  |  |  |  |  | 14/14 |
| **Which specific laboratory tests should be used in patients with clinical suspicion of acute schistosomiasis / Katayama syndrome?**  It is recommended to use a combination of direct and indirect tests to diagnose Katayama syndrome:  - one serological test with high sensitivity and specificity. If serology is initially negative repeat the test after 3-4 weeks from the onset of symptoms and approximately 4-8 weeks after contact with contaminated water to check for seroconversion  - parasitological examination of at least 3 stool/urine samples. If initially negative repeat the test after 4-8 weeks from the exposure.  In case of availability, you may consider using:  - PCR on serum;  - CAA detection on serum and/or urine. |  |  |  |  | 1/14 |  |  | 1/14 | 12/14 |
| **How acute schistosomiasis / Katayama syndrome should be treated?**  -All patients with probable or proved acute schistosomiasis must be treated with an antiparasitic drug associated with steroids administration;  -Empiric treatment with PZQ may be considered before the parasitological confirmation in travellers from endemic countries who presents with possible acute schistosomiasis;  -PZQ dosage for acute schistosomiasis: 40mg/kg/day for *S. mansoni* and *S. haematobium* and 60 mg/kg/ day for *S. japonicum*, both in 2 divided doses the same day for 1-3 days associated with steroids administration (prednisone 25 mg/die or equivalent for 3-6 days with progressive de-escalation during 2-3 weeks). Repeat PZQ in 6-8 weeks when all worms will have developed to adults. |  |  |  |  |  |  | 1/13* |  | 12/13* |
| **CHRONIC SCHISTOSOMIASIS** | | | | | | | | | |
| Statement | LIKERT SCALE | | | | | | | | |
|  | 1 | 2 | 3 | 4 | 5 | 6 | 7 | 8 | 9 |
| **Who should be screened for schistosomiasis?**  - Screening for schistosomiasis is recommended in all subjects, included asymptomatic, who were born or have lived for at least 6 months in endemic countries; |  |  |  |  |  |  | 1/14 | 1/14 | 12/14 |
| - Screening is also recommended in all subjects, independently by their country of origin, who have visited endemic countries even for short periods (e.g., tourists) and who may not exclude freshwater exposure. |  |  |  |  |  |  |  | 2/14 | 12/14 |
| **How to screen for schistosomiasis?**  - Serological tests are the recommended screening tools considering their high sensitivity for detection of schistosomiasis in low-endemicity settings |  |  |  |  |  |  |  |  | 14/14 |
| **When chronic schistosomiasis should be suspected?**  The diagnosis of schistosomiasis should be considered in subjects who present the epidemiological criterion (travel or origin from endemic area) and at least one among the followings:  a) signs and/or symptoms, even if non-specific, affecting the gastrointestinal system (abdominalgia, hepato- and/or splenomegaly);  b) signs and/or symptoms, even if non-specific, affecting urogenital system (e.g., haematuria, dysuria, lower back pain, hemospermia etc.);  c) eosinophilia. |  |  |  |  |  |  |  |  | 14/14 |
| **Which specific laboratory test should be used in patients with clinical suspicion of chronic schistosomiasis?**  A combination of direct and indirect tests to diagnose chronic schistosomiasis is recommended:  - at least one serological test with high sensitivity and specificity  - parasitological examination of at least 3 stool/urine samples  In case of availability, you may consider using:  - DNA detection tests on stool, urine and serum;  - CAA detection on serum and/or urine  Tissue biopsy may be considered in selected case of high diagnostic suspicion when diagnosis may not be confirmed by less invasive methods. |  |  |  |  | 1/14 |  |  | 1/14 | 12/14 |
| **Imaging in patients with chronic schistosomiasis**  Ultrasound of the abdomen is recommended in the following situations:  -symptoms suggestive of chronic schistosomiasis;  -signs suggestive of chronic schistosomiasis (e.g., haematuria, microhaematuria, splenomegaly, history of hematemesis);  -comorbidities (e.g., HBV or HCV infection);  -positive parasitological examination of stool or urine or other direct diagnostic methods such as CAA or DNA detection tests. |  |  |  |  |  |  |  |  | 13/13* |
| Ultrasound in asymptomatic patients affected by chronic schistosomiasis with negative parasitological tests (isolated positivity of serology) may still be offered if economic and organizational resources are available. |  | 1/13* |  |  |  |  |  | 3/13* | 10/13* |
| **When a cystoscopy should be performed in patients with urogenital schistosomiasis?**  -Cystoscopy ± biopsy is recommended in patients with urogenital schistosomiasis if signs such as haematuria or bladder lesions persist >6 months after PZQ therapy;  -Cystoscopy with biopsy can be considered in case of bladder imaging highly suggestive of neoplasia that do not begin to regress after 2-3 months after PZQ treatment;  -Cystoscopy ± biopsy is not recommended in patients with a clinical suspicion of urogenital schistosomiasis since the diagnosis can be obtained through non-invasive tests (such as serology and parasitological test). |  |  |  |  |  |  |  |  | 13/13* |
| **When a colposcopy should be performed in patients with suspected genital schistosomiasis?**  - Colposcopy is recommended in women suspected of FGS if signs and symptoms of genital involvement are present in order to verify differential diagnoses and co-morbidity.  - Colposcopy is recommended in women who complain of genital discomfort even before the diagnosis of chronic schistosomiasis is formulated. A serology test is recommended in case of a suggestive clinical picture |  |  |  |  |  |  |  | 2/13* | 11/13* |
| Colposcopy is recommended in women who complain of genital discomfort even before the diagnosis of schistosomiasis is formulated. A serology test is recommended in case of a suggestive clinical picture. |  |  |  |  |  |  |  |  | 13/13* |
| **Who should receive antiparasitic treatment for schistosomiasis?**  -All patients with probable or proved schistosomiasis must receive the specific antiparasitic treatment (PZQ) |  |  |  |  |  |  |  | 1/14 | 12/14 |
| -Empiric antiparasitic treatment with PZQ may be considered in i) migrants from countries with high prevalence of schistosomiasis in context of public health initiatives; ii) patients with high clinical suspicion of schistosomiasis but without a parasitological confirmation |  |  |  |  |  |  |  | 1/14 | 14/14 |
| **What is the recommended antiparasitic treatment for schistosomiasis?**  -Praziquantel is the first line antiparasitic treatment for schistosomiasis; |  |  |  |  |  |  |  |  | 14/14 |
| -Praziquantel dosage for chronic schistosomiasis: 40 mg/kg/day po in 1 or 2 divided doses the same day for S. haematobium e S. mansoni, 60 mg/kg/day in 3 divided doses the same day for S. japonicum e S. mekongi. |  |  |  |  |  |  |  | 1/13* | 12/13* |
| -Calculate the final dose on patient’s weight and administer divided doses 4-6 hours apart. It is recommended to take the drug with or immediately after a meal to guarantee optimal absorption; |  |  |  |  |  |  |  | 2/14 | 12/14 |
| -In non-endemic areas it is suggested to repeat the daily dosage for three consecutive days for non-pregnant and non-breastfeeding subjects ≥ 4 years old with chronic schistosomiasis; | 1/13* |  |  |  |  |  |  | 2/13* | 10/13* |
| Investigate past history of seizures or skin nodules and clinically rule out neurocysticercosis or ocular cysticercosis before prescribing PZQ. |  |  |  |  |  |  |  | 1/13* | 12/13* |
| **How PZQ should be used in special populations (pregnant and breast-feeding women, children, subjects with HIV, TB and TBI?**  -Pregnancy and breast feeding: praziquantel may be used during pregnancy and breast feeding with standard dosage (over 1 day). In non-endemic settings, the benefit of treating a pregnant woman must always be balanced with the risk of disease progression in the absence of adequate treatment. |  |  |  |  |  |  |  |  |  |
| -Children aged less than 4 years old: PZQ is recommended at standard dosage (over 1 day, off label use of PZQ). It is possible to crush the tablets and give them together with a soft food or drink. |  |  |  |  |  |  |  |  | 13/13* |
| -HIV co-infection: evaluation of drug interactions required. |  |  |  |  |  |  |  |  | 13/13* |
| -Patient with active TB or TBI: administer PZQ prior to initiation of rifampicin therapy in order to avoid sub-optimal treatment for schistosomiasis |  |  |  |  |  |  |  |  | 13/13* |
| **How patients with complicated chronic schistosomiasis should be managed?**  After PZQ treatment, subjects with urogenital or hepato-intestinal schistosomiasis with organ complications should be managed with an individualized and multidisciplinary approach, possibly at a referral centre |  |  |  |  |  |  |  | 1/13* | 12/13* |
| **How patients with chronic schistosomiasis should be followed-up after antiparasitic treatment?**  -In asymptomatic subjects with probable infection in which only serologic data support the diagnosis, no follow-up is recommended. |  |  |  |  |  |  | 1/14 |  | 13/14 |
| -In subjects with eggs of Schistosoma spp. in stool or urine, parasitological monitoring is recommended 2-3 months after treatment with PZQ and, if viable eggs are still detected, a new antiparasitic treatment is recommended. Assessment of eggs viability is important since not viable eggs can be eliminated with excreta over time after parasitological cure. |  |  |  |  |  |  | 1/14 |  | 13/14 |
| -In subjects with hepato-intestinal schistosomiasis and pathological findings at imaging, ultrasound monitoring is recommended 6 months after the end of treatment. The frequency of ultrasound follow up may be modified depending on the severity of the picture and presence of varices. |  |  | 1/14 |  |  |  |  | 1/14 | 12/14 |
| -In subjects with urogenital schistosomiasis with bladder wall lesions, ultrasound monitoring is recommended at 1, 3 and 6 months until lesions disappearance. The persistence of bladder lesions at 6 months after treatment should lead to histological investigation through biopsy to rule out carcinomatous evolution and differential diagnoses. |  |  |  |  |  |  | 1/14 | 1/14 | 12/14 |
| * for some statement one of the internal experts (parasitology specialist) preferred not to express her vote when she felt outside her field of knowledge | | | | | | | | | |
